# Neurofibromatosis Type 1 and the “Elephant Man's” Disease: The Confusion Persists: An Ethnographic Study

**DOI:** 10.1371/journal.pone.0016409

**Published:** 2011-02-09

**Authors:** Claire-Marie Legendre, Catherine Charpentier-Côté, Régen Drouin, Chantal Bouffard

**Affiliations:** Division of Genetics, Department of Pediatrics, Faculty of Medicine and Health Sciences, Université de Sherbrooke, Sherbrooke, Québec, Canada; Alexander Flemming Biomedical Sciences Research Center, Greece

## Abstract

**Background:**

During informal interviews in the course of an ethnographic study on intergenerational dialogue between individuals with neurofibromatosis and their parents, many members of Canadian neurofibromatosis associations stated they continue to be told the condition that afflicts them or their children is the “elephant man's” disease. Today, even though well established clinical criteria make it possible to diagnose and differentiate the two diseases, the confusion between NF1 and the disease of Joseph Merrick, the “elephant man”, persists in both media representations and those of physicians. The objective of this article is to document the persistence of this confusion, to identify the factors that contribute to it, and to identify its impact on the well being of individuals with NF1.

**Methodology:**

Preliminary stages of an ethnographic study.

**Principal Findings:**

Our findings show that some reference sources, past medical training, and print and online news media have all contributed to the persistence of the association between NF1 and the disease of Joseph Merrick, the “elephant man”. Our observations suggest that this misconception can have negative medical, social, and psychological impacts on patients and their families and thus increase the burden of the disease.

**Conclusions:**

Changes of attitude regarding medical teaching and the media could lead to definitively clearing up the confusion.

## Introduction

During informal interviews in the course of an ethnographic study on intergenerational dialogue between individuals with neurofibromatosis and their parents, some members of Canadian neurofibromatosis associations stated to the authors that they continue to be told the condition that afflicts them or their children is the “elephant man's” disease. This puzzled us, because the identification of neurofibromatosis with the condition from which Joseph Merrick, the so called “elephant man”, suffered does not correspond to current medical knowledge.

**Figure 1:**
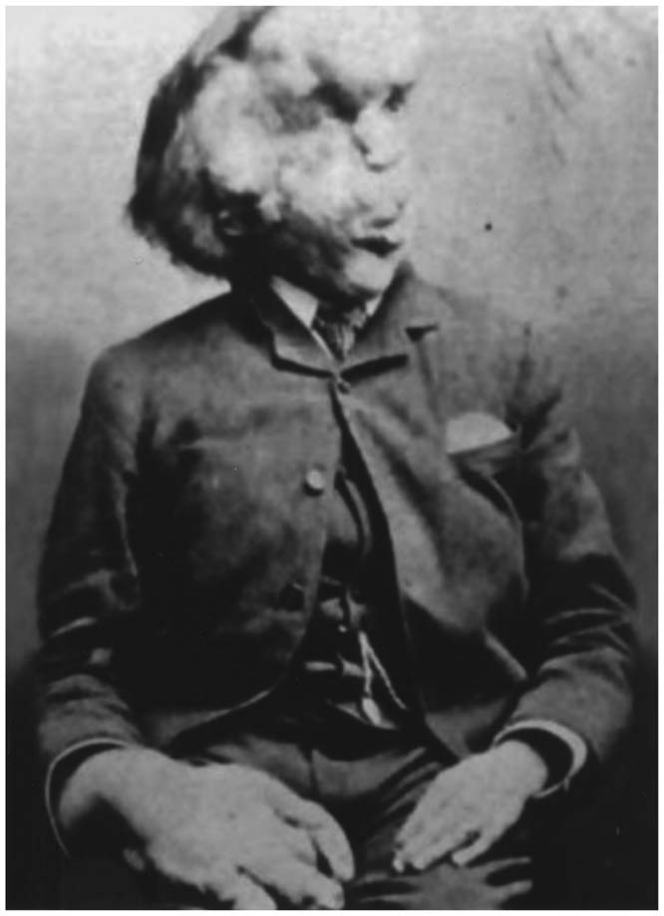
A man who suffered from Proteus syndrome (Joseph Merrick, the “elephant man”). Figure 1 is in the public domain in USA (published before 1923) (see http://fr.wikipedia.org/wiki/Fichier:Joseph_Carey_Merrick.png).

For many years, it was thought that Joseph Merrick, widely known as the “elephant man” (Figure 1), suffered from NF1 [1], [2], [3], [4], [5], [6]. From 1909 on, however, other diagnoses were advanced. At last, in 1986, Canadian geneticists Tibbles and Cohen demonstrated that Merrick was actually afflicted with Proteus syndrome [7].

Today it is known that NF1 is one of the world's most widespread mendelian genetic disorders, with a prevalence of 1 in 3,000. In contrast, Proteus syndrome is a very rare condition, with a prevalence lower than 1 in 1,000,000 (Table 1 [8], [9], [10], [11], [12], [13], [14]) [15]. Furthermore, diagnostic criteria for both conditions have been well defined by the NIH (Table 1), and there exist methods of genetic testing that differentiate reliably between NF1 and Proteus syndrome. In spite of this, the general public, NF1 sufferers themselves, and some health professionals continue to be under-informed about NF1 and to confuse it with Joseph Merrick's disease [12].

**Table 1:** Comparative table of the two disorders' various features*.

NEUROFIBROMATOSIS	PROTEUS SYNDROME
**Prevalence**	
Frequent: 1/3,000–1/4,000 live births worldwide	Extremely rare: <1/1,000,000 live births worldwide
Equally prevalent in males and females	Two males for every female
**Gene**	
NF1, chromosome 17 (17q11.2)	Unknown
**Transmission**	
50% hereditary (autosomal dominant)	Sporadic
50% sporadic (*de novo* mutation)	Postzygotic somatic mutation (embryonic lethal in non-mosaic form; never with diffuse involvement of the entire body)
**Diagnostic Criteria**	
An individual has NF1 if at least two of these criteria are present.	An individual has Proteus syndrome if the three general criteria plus one criterion from category A or two from category B or three from category C are present.
	**General Criteria**
1. Café-au-lait spots (at least 6): Diameter >1,5 cm after puberty; >0,5 cm before puberty	1. Mosaic distribution of lesions
2. Neurofibromas (at least 2 of any types) and/or one or more plexiform neurofibromas	2. Sporadic occurrence
3. Axillary and/or inguinal freckling	3. Progressive course
4. Optic gliomas	**Category A**
5. Lisch nodules (2 or more)	1. Cerebriform connective tissue nevus
6. Characteristic osseous lesion (sphenoid dysplasia, thinning of long bone cortex with or without pseudoarthrosis	**Category B (two required)**
7. First degree relative with NF1	1. Linear epidermal nevus
	2. Asymmetric, disproportionate overgrowth
	3. Specific tumors before second decade
	**Category C (all three required)**
	1. Lipomas or focal atrophy of adipose tissue
	2. Capillary, venous, or lymphatic malformation
	3. Facial features including dolichocephaly, a long face, down-slanting palpebrae, ptosis, depressed nasal bridge, anteverted nares, and open mouth position while at rest
**General clinical manifestations**	
1. High blood pressure	1. Pulmonary abnormalities
2. Scoliosis	2. Renal abnormalities
3. Malignant tumors	3. Brain malformations
**Cognitive and psychological problems**	
1. Attention deficit (with or without hyperactivity) disorder in 40% to 50% of cases	1. Learning difficulties in 20% of cases
2. Learning difficulties in 30% to 65% of cases	2. Mental retardation in 10% to 15% of cases
3. Slight mental retardation in 1% to 8% of cases (no consensus among authors)	3. Psychological consequences of the disease
4. Difficulty forming friendship in childhood	4. Feeling of isolation
5. Impact on quality of life	5. Social stigmatization
6. Difficulty establishing social relationships	6. Courtesy stigma afflicts family members
7. Psychological/psychiatric disorders	
8. Esthetic considerations represent a psychological burden	

*Adapted from Cohen, 1993; Hart, 2005; Biesecker, 2006, 2005, 2002; Turner, 2007; NIH, 1998.

It becomes clear that this is a problematic situation once it is understood that confusing NF1 with Proteus syndrome and using the term “elephant man disease” as a name for both can have serious clinical, social, and psychological repercussions for individuals with NF1 and their families [1], [2], [5], [16]. As we will show in this article, despite Tibbles and Cohen's work and the further knowledge that has been acquired about the two genetic disorders since Tibbles and Cohen, the confusion of NF1 with the disease from which Joseph Merrick suffered continues to be perpetuated in medical and social representations, by current linguistic usage, and in some media reports [1], [16].

Given current medical knowledge and the psychosocial and clinical consequences of confusing neurofibromatosis type 1 (NF1) with the condition suffered by Joseph Merrick, that is, Proteus syndrome, we wished: 1) to document the persistence and extent of this fallacy; 2) to identify certain critical factors that contribute to its persistence; and 3) to evaluate its impact on the health and well being of individuals with NF1 and their family members. To reach these objectives, we had to situate our informants' testimony (and testimonies) in its cultural, social, and medical contexts. Taking an exploratory approach, we opted to begin by examining the written media, because these have a known impact on social representations, and then follow up by investigating the medical community's treatment of the subject, because of the prominent role its members play in patients' lives and their influence on the quality of knowledge transmitted.

In the present article, after presenting a methodological overview, we examine the persisting confusion between NF1 and Joseph Merrick's condition and the forms it takes in the print and online news media. Thereafter, we provide several examples of the impact this misconception can have on the health and well being of individuals with NF1 and their family members. To conclude, we propose some approaches to reducing the negative impact of the confusion between NF1 and the “elephant man's” disease on the lives of patients and their family members and to ensuring patients receive better clinical management.

### Neurofibromatsis type 1 (NF1)

**Figure 2:**
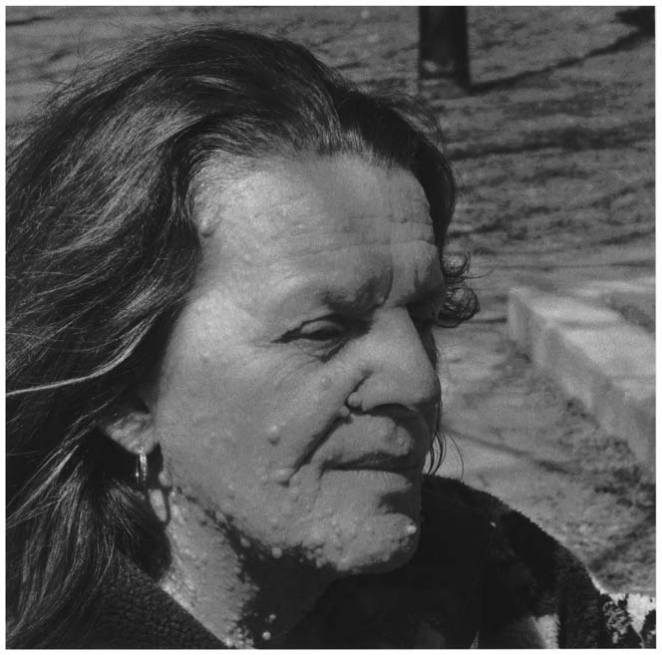
A woman who suffers from NF1. (Photo: Marie-Soleil Lemay-Couture.).

With a prevalence of 1 in 3,000, NF1 is one of the most widespread autosomal dominant diseases in the world. One way of giving an idea of its pervasiveness is to point out that it occurs almost as often as cystic fibrosis (1 in 3,500) [17]. The disease predisposes sufferers to a high risk of both benign and malignant tumours [18]. The diagnostic criteria for NF1 are well known (see Table 1). Since the NF1 gene is expressed in all of the body's cells, its clinical manifestations (Table 1) can be cutaneous, skeletal, ophthalmological, and neurological [19]. NF1 can give rise to, among other things, serious esthetic problems (see Figure 2), learning difficulties, cognitive deficits, and psychosocial problems, which in turn are frequently associated with academic difficulties (see Table 1) [20], [21], [22], [23], [24].

### Proteus syndrome

Unlike NF1, Proteus syndrome is an extremely rare genetic condition, with only 205 recently published cases worldwide, of which only 97 satisfy the diagnostic criteria issued by the NIH in 1998 [15]. The causal mutation seems to occur in a consistently sporadic manner and the subsequent syndrome manifests itself in a mosaic pattern [15], [25]. Contrary to what is the case with NF, no specific genetic mutation has been identified for Proteus syndrome [15]. Proteus syndrome manifests as the excessive and unbalanced growth of multiple tissues and is also characterized by conjunctive tissue calcifications and narrowing and lengthening of long bones [15].

## Materials and Methods

At the time of writing of this article, we are conducting an ethnographic study on the state of intergenerational dialogue between parents and children with neurofibromatosis. The ethnographic methodological approach entails, among other things, observing the individuals and groups being studied in the settings where they interact. In empirical-inductive anthropological studies, the environment is not controlled. Thus it is common for researchers to encounter unforeseen phenomena which, because of their inherent significance, must be taken into account regardless of how closely they relate to the set of issues initially intended to be under study.

This is what occurred during our study on intergenerational dialogue. In the course of conversations conducted in the field, we came face to face with a phenomenon that, while it was unrelated to our research objectives, raised ethical and clinical concerns that struck us as sufficiently fundamental to form the focus of a supplementary study. To avoid confusion, we wish to specify here that it is the results of that supplementary analysis that we are presenting in this article and not the results of the original study on ingergenerational dialogue. But since the methodological context in which we encountered the phenomenon is that of the original study (which is still under way), we present the approach we took to methods in chronological order.

### Initial methodology

For our study on intergenerational dialogue between parents and children with neurofibromatosis, our study design comprises three classical ethnographic investigative methods: 1) critical and comparative analysis of the literature; 2) participant observation; and 3) semi-structured interviews [26].

#### Critical and comparative analysis of the literature

The purpose of the initial analysis of the literature was to understand and analyze the medical, psychosocial, and cultural aspects of neurofibromatosis as discussed to date in the scientific literature. In standard fashion, this stage occurred prior to that of participant observation.

#### Participant observation

Participant observation requires investigators' full physical and intellectual immersion in the setting under study. The participant observation stage of the study on intergenerational dialogue was begun in 2007. It consisted of: 1) over 300 hours of *in situ* observation at events held by NF associations (colloquia, annual meetings, social activities, day-long information sessions, directors' meetings, and so on); 2) a lecture for the general public on the medical, anthropological, and ethical aspects of NF; 3) discussions with association members; and 4) exchanges elicited by our presentations of our research project at the associations' annual meetings. By these means, we gathered important information about NF association members' experience of the disease and the difficulties they encounter in daily life. The observation data and the testimonies gathered from discussions and numerous informal interviews with association members were entered in a field journal. Because the interviews were informal and because they occurred in the context of participant observation, we did not collect sociodemographic data about our informants.

#### Initial study subjects

We participated in nine events (annual meetings, social activities, daylong information sessions, directors' meetings) held by a number of Canadian neurofibromatosis associations. On average, we met about 40 people at each event. For the most part they were patients and patients' family members, but some were health and education professionals who were well informed about problems associated with NF1. We were three researchers in the field; and from 2007 on we gathered several hundreds of confidences and stories, thereby ensuring that data saturation was more than sufficient for the purposes of a qualitative study.

### The emergent phenomenon

Since the presentations we made related to diverse aspects of NF1, people were inclined to speak to us spontaneously about what they were experiencing in connection with the disease. Thus during the participation observation process many NF1 patients or parents of children with NF1 told us of the extent to which the identification of NF1 with the disease of Joseph Merrick, the “elephant man”, had impacted on their lives both medically and psychosocially. This phenomenon was not among the themes we had originally planned to explore. However, in view of its ethical and clinical significance, this emergent issue required its own analysis. For this reason, we undertook an exploratory study aiming to: 1) document the persistence and extent of this fallacy; 2) identify certain critical factors that contribute to its persistence; and 3) evaluate its impact on the health and well being of individuals with NF1 and their family members.

#### Subsequent critical and comparative analysis of the literature

In this context we decided to 1) carry out an analysis of the scientific literature that was tailored to this new issue about the confusion between Proteus syndrome and NF1; and 2) pinpoint articles in the print and online news media from 1988 to 2009 in which NF1 was identified with the condition suffered by Joseph Merrick, the so called “elephant man”.

The review of the literature that related specifically to the phenomenon discussed in the present article was carried out using databases specialized in medicine (PubMed, MEDLINE, EMBASE, Web of Science), psychology (PsycARTICLES, PsycINFO, ERIC, CAIRN, Academic Search Complete), social sciences (CBCA Complete, CINAHL, FRANCIS, ProQuest Dissertations and Theses, Social Work Abstracts, SocINDEX), and philosophy (Repère, Eureka.cc, Philosopher's Index). We used the following search terms in both French and English: “neurofibromatosis type 1”, “NF1”, “von Recklinghausen disease”, “Proteus syndrome”, “elephant man”, “elephant man disease”, “confusion”, and “misconception”.

We looked for articles published from 1909 to the present, since it was in an article by Parkes Weber published in 1909 that the premise that Joseph Merrick suffered from NF1 was mentioned for the first time. Another factor we looked for was the number of articles published before and after 1986 that maintained the confusion, since Tibbles and Cohen's research results clearing up the confusion were published in 1986.

As for online media articles, we chose 1988 as the starting point because it is in that year that articles began to appear in the media pointing out that NF1 does *not* have anything to do with the disease Joseph Merrick had. We also took into account certain print and electronic reference sources available to the general public in 2008 and 2009.

#### Subsequent participant observation

Once association members had told us that they continued to suffer from the confusion between NF1 and the disease Joseph Merrick had, we were sensitized to the suffering created by this situation. As of 2008 we began doing poster presentations to explain the difference between the two diseases at regional, national, and international scientific events (see Table 2). It was at this time that we also decided to gather the comments made by physicians and students who stopped at our poster presentations to discuss how they themselves or others whom they knew had fallen prey to the confusion between NF1 and Proteus syndrome.

**Table 2:** Scientific Events at Which Presentations Were Made on Psychosocial and Ethical Issues Surrounding Neurofibromatosis.

Scientific Event	Year	Country	Context	People We Met	Number of People Who Gave an Opinion
2iéme journée scientifique de l'Axe mère-enfant (Second Annual Science Day of the Mother-Child Center), Centre de recherche clinique Étienne-Le Bel, CHUS, Sherbrooke, Quebec	2008	Canada	A science day at Université de Sherbrooke	Physicians, medical students	6
6ième journée scientifique du Département de Pédiatrie (Sixth Annual Science Day of the Department of Pediatrics), CHUS, Sherbrooke, Quebec	2008	Canada	A science day at Université de Sherbrooke	Physicians, medical students	7
50th Annual Meeting of the Club de recherches cliniques du Québec	2008	Canada	National congress	Physicians, medical students	6
58th Annual Meeting of the American Society of Human Genetics	2008	USA	International congress	Physicians, medical students	5
51st Annual Meeting of the Club de recherches cliniques du Québec	2009	Canada	National congress	Physicians, medical students	5
Les conférences de la Relève Gilles-Dupuis, Axe mére-enfant (Relève Gilles-Dupuis Speaker Series, Mother-Child Center), Centre de recherche clinique Étienne-Le Bel, CHUS, Sherbrooke, Quebec	2010	Canada	A science day at Université de Sherbrooke	Physicians, medical students	15
Assises de génétique humaine et médicale (Congress on Human and Medical Genetics)	2010	France	International congress	Physicians	5
TOTAL					**49**

From that point on, we noted such comments in our field journals. As was mentioned above in connection with participant observation, since we were engaging in a process of observation, we did not collate any sociodemographic data about the people we spoke with. In any case, these data would not have been useful in the context of an exploratory study which had as one of its aims to learn whether the confusion in question persists among physicians and medical students and if so, why. We were not seeking to learn the frequency of this phenomenon in the target population. The kind of study with which such knowledge could be obtained can only be conducted once the problem has been identified, which is the scientific purpose served by exploratory studies.

#### Research subjects

In this context, then, we gathered comments from 49 subjects (Table 2) who were medical students or physicians, with ages, specializations, genders, and countries of origin undifferentiated. The criteria for inclusion were simply that our informants made comments on the fact that the confusion persists or that they themselves had been subject to it and their ideas about the causes of the confusion.

#### Ethical approval

The Draft 2nd Edition of the Canadian Tri-Council Policy Statement states: “*Qualitative research approaches involving a community, group or population of interest (e.g., marginalized or privileged groups) follows a process of prior dialogue, exchanges and negotiation of the research, which precedes the formal data collection involving human participants*” and “*preliminary activities may include note taking, scribbling, diary writing, and observation made long before the researcher has any inkling that these would turn into formal research projects. These types of preliminary activities are not subject to REB review*”. (Article 10.6, chapter 10, Draft 2nd Edition of the Tri-Council Policy Statement: Ethical Conduct for Research Involving Humans, 2008).

The data presented here emerged from these preliminary activities: literature review, participant observation, and informal discussions with members of partner associations. The obtaining of research ethics board approval is in process for the next stage of the original study (semi-structured interviews).

Semi-structured interviews: This article will not discuss the semi-structured interviews, because those relate to the original study's theme of intergenerational dialogue. Those interviews will, however, contain questions about the confusion of NF1 with the disease of Joseph Merrick, the “elephant man”.

## Results

Since these empirical data obtained from participant observation results had raised the ethical question of the confusion between NF1 and Joseph Merrick's disease, it became necessary for us to review and analyze documents on this issue. In what follows, we present the results of our analysis of scientific articles and print and online media publications as well as results from participant observation.

### NF1 and Proteus syndrome in the scientific literature

The scientific literature we reviewed and analyzed consisted of 26 articles on NF1 that used the words “elephant man”. Of these, as expected, all those dating form 1909 to 1986 said that Joseph Merrick suffered from NF1 [27], [28], [29]. After 1986, some authors persisted in the confusion. Hostamisligil and Seward actually question Tibbles and Cohen's diagnosis [30], [31], [32]. But for the most part, the scientific articles published later than 1986 no longer perpetrate the confusion [1], [3], [4], [16], [33], with three of them explicitly condemning it [1], [16], [34].

### The confusion between NF1 and the “elephant man's” disease as found in print and online news media

In 1988, two years following the publication of Tibbles and Cohen's findings [7], the American newsmagazine *Newsweek* reported, in a brief news item called “What the Elephant Man really had”, that Joseph Merrick had not suffered from NF1 [35]. Despite this emergence of the information into the news media, numerous American newspapers went on associating NF1 with Joseph Merrick, the “elephant man” [36], [37], [38], [39], [40]. In response, in a letter to the editor that appeared in the *New York Times* in 1989, Joan Rudd Engel, a past president of the National Neurofibromatosis Foundation, explained how distressing this confusion could be to NF1 patients: “As brave and inspiring as [Joseph Merrick] has proved to be, the identification with him for the many with neurofibromatosis proved frightening and painful to them and was also inaccurate. It is in the interest of the many, many thousands of people with neurofibromatosis to see further comparison with the Elephant Man ended.” [41].

A decade later, American anthropologist Joan Ablon was the first researcher to decry the continued association of NF1 with the “elephant man's” disease in the media [1]. Nevertheless, the confusion did not cease. For example, when the first partial face transplant was performed in France in 2007, French newspapers [42], [43], [44] and some American [45] and Canadian [46] media outlets identified the patient's disease, which was in reality NF1, with Joseph Merrick's condition. Passages in the French media include “The patient suffered from von Recklinghausen's disease [an alternate name for NF1], an incurable disease that disfigures the face, as in the case of the hero of the movie *The Elephant Man*.” [43] The only media outlet to correct the misrepresentation was *ABC News*, which later reported that Joseph Merrick had not had NF1 but Proteus syndrome [47]. Media items discussing that face transplant since then have continued to identify NF1 with the “elephant man's” disease [48], [49], [50], [51]. As of 2008, the confusion between NF1 and Joseph Merrick's condition could still be found on Wikipedia, and as of July 2009 the Dictionary.com entry for the term “elephant man's disease” stated that it is NF1 and that it is so called because Joseph Merrick suffered from it [52].

### The confusion between NF1 and Joseph Merrick's disease in the medical world

Even though the clinical differences between NF1 and Proteus syndrome are known, and despite the diagnostic methods available nowadays, the confusion persists not just in press representations but also in those of physicians. A few of our informants' stories of their experiences will suffice to illustrate this. For instance: “During dinner with friends, the conversation came around to the fact that my nephew had just been diagnosed with NF. A doctor who was present told me it was what the “elephant man” had had” (Érika, aunt of a child with NF1, Field Notes, 2008). (In order not to weigh down the text, we only quote one interview excerpt for each of the points raised by the study subjects. But the excerpts have been chosen with a view to reflecting the perspective of the largest possible number of people.) A comment of this kind provokes great anxiety and raises many questions: In this informant's place, one might wonder, “Do his parents know? Should I tell them – and what *can* I tell them? Will my nephew be able to go to school and take part in sports? What will he look like?” In confirmation of this, an informant who is a physician well informed about the difference between the two diseases expressed dismay at the fact that “so much ignorance exists around the subject of NF1” (Paul, physician, Field Notes, 2008).

In a clinical context, given the hierarchical relationship between physicians and patients, the association between NF1 and Proteus syndrome can have important psychological and medical repercussions. One patient told us the following in explaining that she was told more than once that she has the “elephant man's” disease: “The first time was when I was 15. The first neurologist who followed me told me I had the ‘elephant man's’ disease. Even the family physician who followed me between ages 15 to 25 often said to me, ‘Don't forget you have the ‘elephant man's’ disease, you could end up looking like him’. I didn't find this funny. The only thing he knew about the disease was what you could find in outdated medical encyclopedias. I don't resent him for this, though. It's thanks to him that I was diagnosed” (Kathy, woman with NF1, Field Notes, 2009). Another informant told us, “Hearing a doctor tell you in the ER (Emergency Room) that your child has the ‘elephant man's’ disease is traumatic” (Joyce, mother of a child with NF, Field Notes, 2008).

It is usually from their associations or from specialists familiar with NF1 that patients learn they don't have the condition from which Joseph Merrick suffered. Sometimes patients or family members have to inform physicians of this: “I actually had to explain to [the doctor] the difference between NF1 and Proteus syndrome. That was so very frustrating to hear that from a doctor in 2008” (Joyce, woman with NF1, Field Notes, 2009). Testimony to this effect from our informants confirms what Radtke et al. wrote in 2006: “Many families continue to be misinformed that NF1 is the ‘Elephant Man Disease’.” [16].

Since many physicians currently practicing were trained before the publication of Tibbles and Cohen's article in 1986, we can expect the confusion between NF1 and Proteus syndrome to continue for some years. During our poster presentations, many physicians we spoke to made comments like that of Georges: “When I was a resident, we were taught that Joseph Merrick suffered from NF1” (Georges, MD, PhD, Field Notes, 2008). Medical students also often made comments *about NF1* like this one: “Hey, I know about that disease! It's the disease the ‘elephant man’ had; we talked about it in class” (Joan, medical student, Field Notes, 2009). Finally, *Stedman's Medical Dictionary* provides two definitions of “elephant man's disease”, one being that it is a synonym for Proteus syndrome and the other that it is a colloquial way of referring to NF1 [53].

A final point is that some physicians actually view the confusion as yielding benefits in the form of publicity for NF1: “If it brings NF1 to the attention of people around the world and makes it clear that it's a serious disease, that's a good thing” (John, MD, PhD, Field Notes, 2008). People who live with NF1 do not take this view. “We've been fighting for years to put an end to this confusion…. All it does is reinforce prejudices about the disease and intensify the sufferings of patients and their family members” (Gloria, mother of a child with NF1, Field Notes, 2007).

### The impact of the confusion between NF1 and Proteus syndrome on the health and well being of individuals with NF1 and their family members

As we have seen, some reference sources, past medical training, and the print and online news media have all contributed to the persistence of the association between NF1 and the disease of Joseph Merrick in representations by the general public and health professionals. The viewpoint of patients and their family members is that this confusion distorts how people perceive individuals with NF1. Moreover, it is known that NF1 sufferers experience difficulty establishing social ties and developing good self-esteem [54], [55]: having their condition identified as the same disease as the “elephant man” had or given the name “elephant man disease” can only deepen this difficulty.

A press release published in 2001 by the board of directors of Quebec's neurofibromatosis association provides a good overview of the impact of this confusion on patients and their families: “The Board of Directors of the Association de la neurofibromatose du Québec (ANFQ) is concerned about the fact that a few members of the medical profession continue to associate neurofibromatosis with elephant man disease…. The stakes of this identification are considerable: considering the imaginary stigma created by the publicity given to the elephant man, it is a tragic event in a family to learn that one or more of its members, with neurofibromatosis, allegedly suffer from *elephant man disease*, that is, that their condition could degenerate to the point of being a monster. As a result, people with the disease and their close ones can become profoundly affected by the idea of such an eventuality.” [5] (Provided in English on the Association's web site; italics in the original.)

For those with NF1, believing they have Joseph Merrick's condition or knowing others believe this heavily compromises their hopes of a normal social life, employment that interests them, an enduring couple relationship, and raising children [56], [57]. When they learn that 50% of NF1 cases are inherited and that the condition is genetically dominant, they are likely, if they believe the condition they are at risk of transmitting is Joseph Merrick's condition, to dread that their children will look like the “elephant man”. A misunderstanding of this kind influences the process of arriving at decisions about having offspring: “When I was diagnosed, the physician told my husband and me that it would be best if I had my tubes tied, because all our children would have the disease. That's what I did, and I was stunned to learn later that what he had said wasn't true. First of all, the disease isn't that bad; and there were other solutions” (Béatrice, woman with NF1, Field Notes, 2007).

Furthermore, even though NF1's prevalence is similar to that of cystic fibrosis, one of the main problems faced by individuals with NF1 is to find a physician who can provide clinical management during their adulthood (all of the affected persons we have met mentioned this problem). NF1 sufferers often require referrals to specialists of several different kinds (geneticist, dermatologist, neurologist, psychologist and ophthalmologist), so their family physician must be well informed about their disease. And finally, the prospect of becoming like the “elephant man” needlessly complicates clinical management and does harm to the mental health of NF1 patients. We now know that the disease's visibility can have an impact on the quality of life of NF1 sufferers [58] and that there is a significant association between the disease's visibility and psychiatric morbidity [57], [59].

## Discussion

We have seen that, despite the demonstrable damaging effects of the failure to differentiate between NF1 and Joseph Merrick's condition, despite the known major differences between the two conditions, and despite the diagnostic methods available today, the confusion between NF1 and Proteus syndrome persists. Testimonies from the members of Canadian neurofibromatosis associations and our findings from a literature review and field observations suggest that two factors chiefly contribute to this situation: 1) many news medias' failure to reflect more accurate recent knowledge about the “elephant man's” real condition; and 2) the association between NF1 and the “elephant man's” disease made by numerous physicians.

In connection with both factors, our data suggest that continuing to misleadingly use the term “elephant man disease” as a name for NF1 or to erroneously identify NF1 with Joseph Merrick's condition contributes to a climate of inadequate knowledge about the condition, and this can only harm the health and quality of life of patients and their family members. Thus the whole issue raises major professional-ethics, bioethical, and socioethical questions.

From the point of view of professional ethics, physicians have the duty to consider their own capabilities and limitations in exercising their profession. This means referring patients with suspected NF1 to colleagues with appropriate expertise or to specialized NF clinics. In patients' best interests, physicians must refrain from giving information about a disease about which they have little knowledge.

Speaking bioethically, if the medical community were better informed about NF1, the feelings of dread experienced by patients and their families would be reduced. Patients would also receive better clinical management of their cases. To strive for these improvements corresponds to the bioethical principle of beneficence: 1) do no harm, 2) maximize possible benefits, and 3) minimize possible harms. For the sake of patients and their families, it is essential to work towards definitively clearing up the confusion.

Socioethically speaking, confusing NF1 with Proteus syndrome or using the same term, elephant man disease, to designate both affects patients' self-perception and their hopes for a job, a family, and a normal social life. The confusion further stigmatizes them and adds to the disease's own difficulties (Table 1). As well, there are approaches to teaching-and-learning especially suited to helping children with NF1 that can be adopted in school settings [60], [61]. If a lack of awareness about NF1 results in children being deprived of these approaches, they could needlessly suffer major negative impacts on their cognitive and psychosocial development.

### Conclusion

Given the nature of the problems we have just discussed, it could take no more than a few changes of attitude by the medical and scientific communities to make significant improvements. Physicians and scientists could send letters with corrective information to those media, publishers, and web sites that associate NF1 with the “elephant man” or refer to it as elephant man disease. It is noteworthy that we found no instances of the confusion between NF1 and Joseph Merrick's disease in the *New York Times* subsequent to the appearance of the letter from the National Neufibromatosis Foundation's past president [41].

NF1 specialists in various fields could take a more direct part in knowledge transfer and physicians' continuing education. Thus family physicians and pediatricians who have little familiarity with NF1 would learn that they can refer their patients with NF1 to NF clinics [2], [16], [60] and such specialists as geneticists, neurologists, and dermatologists. Patients and their family members would benefit from better tailored clinical management of their cases, perhaps even optimal management. This would include having access to better information [16], [19], appropriate genetic counseling [62], and psychological support from an early age [63], as well as receiving follow-up for learning disorders specific to NF [12], [64]. The literature shows that NF1 sufferers who are referred to specialists able to treat their physiological, psychosocial, and cognitive problems can look forward to significant improvements in their health, quality of life, and ability to integrate socially [12], [60].

On a different note, in the school setting, it is important for teachers to be informed that the main difficulties faced by children with NF1 are not related to intelligence but to learning problems and attention disorders [20], [65]: teachers could learn of the various teaching-and-learning approaches that exist to palliate these children's and teenagers' learning challenges and psychosocial fragility [57].

Finally, we hope this article will contribute in some small degree to showing the importance of the roles of the media and physicians in transferring knowledge about NF1 and thus in improving the conditions of life and health of people with NF1 and their families.
