# Correction: Neurofibromatosis Type 1 and the “Elephant Man's” Disease: The Confusion Persists: An Ethnographic Study

**DOI:** 10.1371/annotation/91535cef-2b2e-44a2-b34e-8a48863ab97d

**Published:** 2011-04-27

**Authors:** Claire-Marie Legendre, Catherine Charpentier-Côté, Régen Drouin, Chantal Bouffard

The abstract of this article is similar to the summary of a research letter by the same authors published in American Board of Family Medicine. The authors would like to amend the abstract to read:

Background: In 1986, two Canadian geneticists had demonstrated that Joseph Merrick, better known as the Elephant Man, suffered from the Proteus syndrome and not from neurofibromatosis type 1 (NF1), as was alleged by dermatologist Parkes in 1909. Despite this and although the two diseases differ at several levels: prevalence, diagnostic criteria, clinical manifestations and transmission, the confusion between NF1 and the "elephant man's" disease continues in medical and social representations by current linguistic usage, and in some media reports. With this article, we want to 1) document the persistence and extent of this fallacy, 2) identify certain critical factors that contribute to its persistence, and 3) evaluate its impact on the health and well being of patients with NF1 and their family members.

Methodology: Participant observation in the course of an ethnographic study on intergenerational dialogue between individuals with neurofibromatosis and their parents - Analysis of the scientific literature and of pinpoint articles in the print and online news media.

Findings: Our findings show that because physicians have little knowledge about NF1, several print and online news media and a lot of physicians continue to make the confusion between NF1 and the disease the "elephant man". This misconception contributes to misinformation about the disease, feeding prejudices against affected patients, exacerbating the negative impacts of the disease on their quality of life, their cognitive development, their reproductive choices, as well as depriving them of proper care and appropriate genetic counseling.

Conclusion: If family physicians and pediatricians were properly informed about the disease, they could refer their patients with NF1 to NF clinics and to specialists. Thus, patients and their family members would benefit from better-tailored clinical management of their cases, perhaps even optimal management.

