# Correction: Neurofibromatosis Type 1 and the “Elephant Man's” Disease: The Confusion Persists: An Ethnographic Study

**DOI:** 10.1371/annotation/7647352b-6815-480e-b37f-940f21c47058

**Published:** 2011-04-27

**Authors:** Claire-Marie Legendre, Catherine Charpentier-Côté, Régen Drouin, Chantal Bouffard

The first paragraph of the Conclusion section of the article should have included a citation to the following publication in American Board of Family Medicine. 66. Legendre CM, Charpentier-Cote C, Drouin R, Bouffard C (2011) Neurofibromatosis type 1: Persisting misidentification with "the Elephant Man" disease. J Am Board Fam Med 24: 112-114. 

